# Medical and biomedical research productivity from Palestine, 2002 – 2011

**DOI:** 10.1186/1756-0500-6-41

**Published:** 2013-02-02

**Authors:** Waleed M Sweileh, Sa’ed H Zyoud, Ansam F Sawalha, Adham Abu-Taha, Ayman Hussein, Samah W Al-Jabi

**Affiliations:** 1 College of Medicine and Health Sciences, Department of Pharmacy, An-Najah National University, Nablus, Palestine

**Keywords:** Research, Productivity, Scopus, Medical and biomedical research, Palestine

## Abstract

**Background:**

Medical research productivity reflects the level of medical education and practice in a particular country. The objective of this study was to examine the quantity and quality of medical and biomedical research published from Palestine.

**Findings:**

Comprehensive review of the literature indexed by Scopus was conducted. Data from Jan 01, 2002 till December 31, 2011 was searched for authors affiliated with Palestine or Palestinian authority. Results were refined to limit the search to medical and biomedical subjects. The quality of publication was assessed using Journal Citation Report. The total number of publications was 2207. A total of 770 publications were in the medical and biomedical subject areas. The annual rate of publication was 0.077 articles per gross domestic product/capita. The 770 publications have an h-index of 32. One hundred and thirty eight (18%) articles were published in 46 journals that were not indexed in the web of knowledge. Twenty two (22/770; 2.9%) articles were published in journals with an IF > 10.

**Conclusions:**

The quantity and quality of research originating from Palestinian institutions is promising given the scarce resources of Palestine. However, more effort is needed to bridge the gap in medical research productivity and to promote better health in Palestine.

## Findings

Palestine is an Arab region located in the Mediterranean area. In 1993, following the Oslo peace accords, the Palestinian national authority (PNA) has been established on parts of the West-Bank of Jordan River and Gaza strip. The Palestinian territories have a total population of approximately 4 million inhabitants and a Gross Domestic Product (GDP) of 4 billion USD [1]. Currently, there are 11 universities, 1 distance education university, 12 university colleges, and 19 community colleges in Palestine. Most of these universities were established and developed after 1967.

Palestine is a new state with scarce resources. Therefore, financial support for research in Palestine is limited. Research activities are highly encouraged at the institutional levels and are linked to academic promotion. Some academic institutions in Palestine have launched peer reviewed medical journals to publish articles of Palestinian researchers. Furthermore, the “Publish or Perish” concept to sustain or promote one’s career is mounting pressure on medical faculty members to conduct and publish research.

Several studies have been published about the quantity and quality of biomedical publications in Arab countries [2-7]. However, none was published in Palestine. Therefore, this study was conducted to analyze the quantity and quality of medical and biomedical publications during the period 2002–2011. Such study will lead to better health outcomes, practice, planning and management for the next decade [8-10]. In addition, the momentum for research activity needs to be maintained through continuous analysis of publication from researchers in the country to provide feedback to academic institutions and education planners.

## Methodology

Comprehensive online search was performed in the SciVerse, Scopus which is one of the world’s largest abstract and citation databases of peer-reviewed literature. Scopus contains 41 million records and covers nearly 18,000 titles from 5000 publishers worldwide and provides 100% Medline coverage [11]. The key word entered in Scopus to accomplish the objective of this study was “Palestine” as a country affiliation or “Palestinian” as an affiliation. The keyword “Palestinian” was used because some authors use the phrase “Palestinian Authority” or “Occupied Palestinian Territory” as an affiliation. The study period was chosen to be from January 1, 2002 till December 31, 2011. Fields of selected research were: health sciences, life sciences, social sciences and physical sciences. The data was refined using the limit function to limit research to medicine, biochemistry/molecular biology/genetics, microbiology/immunology, pharmacology/toxicology/pharmaceutics, psychology, health professions, neuroscience, nursing and dentistry. Data from Scopus were exported to Excel then to IBM® SPSS® Statistics 20 software package (IBM, New York, USA) for analysis and graphics. The raw results were normalized by the average of the population and the GDP and GDP/capita.

The *h*-index for the collected data from Scopus was presented. The *h*-index represents the number of citations received for each of the articles in descending order and the h-graph measures the impact of a set of documents and displays the number of citations per article. The journal’s impact factor was evaluated using the Journal Citation Report (Web of Knowledge); (JCR) 2011 science edition by Thomson Reuters (New York, NY, USA).

## Results and discussion

**Figure 1:**
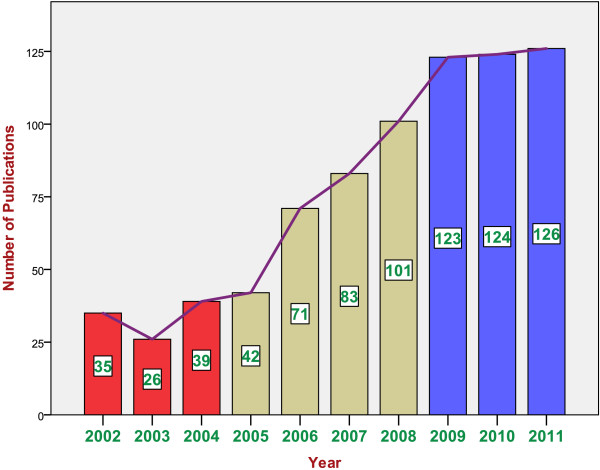
Year-wise distribution of medical and biomedical publications from Palestine from years 2002–2011.

**Figure 2:**
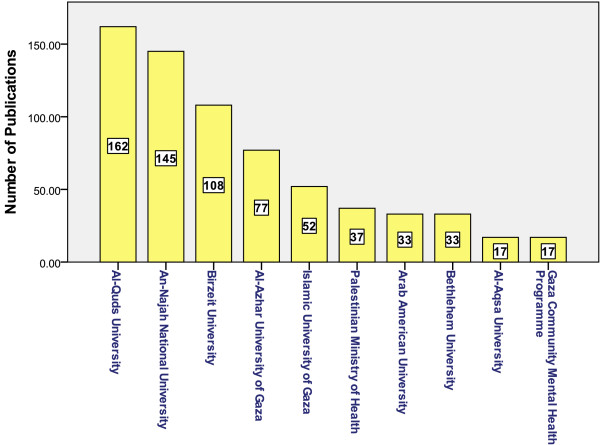
Contribution of various institutes in Palestine to medical and biomedical publications from years 2002–2011.

A total of 2207 publications were retrieved using Scopus. After refining the results, 770 publications were retrieved in the medical and biomedical field giving an average of approximately 80 articles/year or an annual rate of 0.077 per GDP/capita. Figure 1 shows the distribution of publications by year from 2002–2011. It is evident that the number of medical and biomedical publications steadily increased over the last decade. The quantity of publication has increased 4 folds from 2002 to 2011. The increase in the quantity of publication has almost stabilized in the last 3 years of the study period. Figure 2 shows the contribution of various institutions to medical and biomedical publications. Cumulatively, Al-Quds University ranked first (162; 21%) in the total number of publications followed by An-Najah National University (145; 18.8%), Birzeit University (108; 14%), Al-Azhar University – Gaza (77, 10%) and Islamic University – Gaza (52; 6.7%). Cumulatively, original research articles constituted the majority (656; 85.2%) of all publications, followed by review articles (48; 5.5%) and letters to the editor (27; 3.5%). Other types of publications included conference papers, editorials and notes. The total number of citations for the 770 articles was 5538, an average of approximately 7 citations per article. Of the 770 documents considered for the *h* index, 32 have been cited at least 32 times. The 770 articles were published in 158 different peer reviewed journals. Although the number of citations for certain publications might differ from one search engine to another, Scopus search engine remains the best available tool to analyze and track citations and compare citations among different research groups and different institutions.

Publications from Palestine most commonly appeared in Eastern Mediterranean Health Journal (EMHJ) (36; 4.7%) followed by lancet (16; 2%), Pakistan Journal of Medical Sciences (PJMS) (12; 1.5%), Chemical Biology and Drug Design (11; 1.4%) and Tetrahedron Letters (11; 1.4%). The domain of articles published in EMHJ and Lancet was in public and environmental health. Collaboration with international authors indicated that most common collaboration was with Israeli scientists, followed by Americans, German and British. The 770 articles were published in 158 different journals. One hundred and thirty eight (18%) articles were published in 46 journals that were not indexed in the web of knowledge. There were 22 (2.9%) articles published in journals with an IF >10.

There has been a noticeable recent interest in analyzing medical and biomedical publications produced by individual countries [12-15]. Publications reflect the development of medical education, research and health services. There are several scientific search engines to perform this analysis such as Pubmed, Web of Knowledge, Scopus, Google Scholar and others. A study that compared Pubmed, Scopus, Web of Knowledge and Google Scholar have found that PubMed remains an important resource for clinicians and researchers while Scopus covers a wider journal range and offer the capability for citation analysis [16-20]. Furthermore, Scopus also offers author profiles which cover affiliations, number of publications and their bibliographic data, references, and details on the number of citations each published document has received. However, Scopus does not have complete citation information for articles published before 1996. In this study, Scopus was used because publication in journals that are not indexed in Pubmed is common among researchers in Palestine.

Several recent studies assessed research outputs in different Arab countries and compared their productivity to other neighboring countries [6,16,21-29]. Unfortunately, these studies did not include Palestine in the comparison. Furthermore, most of the studies published in Arab countries have used Pubmed as a search engine to investigate productivity. However, one study from Syria has used Scopus methodology to investigate productivity in research and found a total of 458 publications from 1980 – 2011 with an *h* index of 24 [30]. This is relatively lesser than that reported in our study.

Our study is the first detailed analysis of the research publication output from Palestine. Despite the financial crisis and political difficulties in Palestine, the research output during the past 10 years is promising. It was noteworthy that some Palestinian authors have succeeded in publishing in high quality journals like Lancet. International collaboration and have helped the Palestinian researchers to publish in journals with high impact factor. In a comparative study by Benamer and Bakoush, the authors have found that the total number of publications in high impact journals (2001 – 2005) from 16 Arab nations was 83 compared with 412 from 3 other non-Arab nations in the Middle East [23]. Furthermore, the authors of that comparative study have found that the annual publication rate per GDP/capita in the 16 Arab countries did not exceed 1 except for Egypt. Our results regarding annual publication rate per GDP/capita from Palestine seem to be within the range obtained from most Arab countries.

Palestinian medical education and research institutes are relatively young, having been started in early 1980s. The first medical school has been established in late 1990 at Al-Quds University – Abu dis. Currently there are 4 medical schools in Palestine, 2 in west bank and 2 in Gaza strip. There are also 4 schools of pharmacy and similar number of colleges of nursing and allied health sciences. Medical schools are expected to lead biomedical research and publication. It was not surprising that the leading universities in West – Bank are going neck to neck in the number of medical and biomedical publication. It was also not surprising that institutes in Gaza Strip were lagging behind in terms of medical/biomedical publications given the military and economical instability in Gaza strip.

As expected, journals in the field of public health were the most commonly encountered in medical publications from Palestine, particularly from Birzeit and Al-Quds Universities and to a lesser extent from other academic institutions. Public health research is easier to perform and is highly needed in the newborn state of Palestine to establish the baseline health data. However, medical journals in the field of molecular biology, genetics, immunology, and biotechnology were not very commonly encountered. This is not surprising in view of the lack of serious funds for experimental and molecular research activity. The second most common topic encountered in publication was pharmacy in general and pharmacology/toxicology and clinical pharmacy in particular. Pharmacy publications were produced mainly from An-Najah National University. The extensive publication in pharmacy/pharmacology/clinical pharmacy domain from An-Najah National University is attributed to the establishment of the faculty of pharmacy at An-Najah National University since early 1990. Academic institutions and government should encourage researchers to publish in high quality journals and to address research topics that can promote medical practice in Palestine. In this regard, there were only 4 publications from Palestine that addressed professional issues like pharmacy practice and future prospectives and challenges of pharmacy profession in Palestine as well medical practice under occupation.

This study has few limitations. First, publications from local and regional journals that are not indexed in Scopus were not included in the analysis. There is only one local peer reviewed medical journal in Palestine which has been launched in 2011. Unfortunately, this journal is not indexed in Scopus or Pubmed. Furthermore, none of the academic institutions in Palestine have an electronic database posted on their website about their research productivity in the medical and biomedical field for the past decade to gain more insight and to compare with data collected through Scopus. Therefore, it is possible that the number of publications analyzed in this study did not exactly represent all medical publications from academic institutions in Palestine. The best way to overcome such a problem is to do an extensive manual research in all academic and non academic institutions in Palestine about their research productivity in medical and biomedical field. Such manual search needs collective work that must be headed by the Palestinian National Authority, particularly the ministry of higher education. Second, publications by Palestinians working abroad were not included as these publications would be difficult to identify, as well as being affiliated with the institutions where they were produced. Third, there are some international journals that do not recognize Palestine as separate country and publications from Palestine may be affiliated with Israel as a country. Therefore, some publications from Palestine might be missed from our analysis. However, the fact that we used the key words Palestine or Palestinian Authority will minimize this error. Some search engines do not recognize Palestine as a country, but Scopus search engine do recognize Palestine as a separate country which strengthened our claim of research productivity in Palestine and minimized the mix up between Israel and Palestine. In addition, our manual review of each publication and the affiliation of each author in the article made us sure that all publications truly came from Palestine. Even those publications in which the name of an Israeli institution came up had at least one author with a Palestinian affiliation. Despite all this, the data collected and analyzed for this study do represent an approximate estimate of research productivity in Palestine during the past decade and do reflect the differences in research quantity and quality among the different institutions in Palestine.

## Conclusion

Finally, this paper’s main goal is to direct attention and to open doors for a scientific discussion among Palestinian medical professionals and academics. The present data shows promising rise and a good start for medical and biomedical publication from Palestine. Part of this promising rise is due to international collaboration. Therefore, academic institutions in Palestine are advised to strengthen research collaboration with international researchers. The public health aspect of most publications from Palestine is suitable for regional journals rather than international journals with high impact factor. The lack of funding and research infrastructure in Palestine is also a major obstacle for producing research papers suitable for high impact journals. Finally, it is recommended that researchers in Palestine need to select and focus on journals with good scientific reputation for their future publications.

## Availability of supporting data

None.

## Abbreviations

GDP: Gross domestic product; IF: Impact factor.

## Competing interests

The authors have no financial or non-financial competing interests.

## Authors’ contribution

WS: Idea, Analysis and writing the manuscript. SZ: Data analysis, graphics and critique. AS: Literature Review. AA: Literature review and writing the manuscript. AH: critique and thought. SA: critique and thought. All authors read and approved the final manuscript.

## Authors’ information

The corresponding author, Waleed M. Sweileh, is a Professor of Clinical Pharmacology and Pharmacy at the college of Medicine and Health Sciences of An-Najah University, Palestine. He has published more than 70 publications in all fields of medicine and pharmacy in local, regional and international journals. He has a long standing experience in academia in different academic institutions in Palestine.

## References

[B1] World-Bank http://data.worldbank.org/country/west-bank-gaza

[B2] Abu-Zidan F Quantity and quality of research from the Gulf Corporation Council countries Saudi Med J 2001 22 11 1040 1041 11744987

[B3] Afifi M Egyptian biomedical publications in PubMed, 1996–2005 J Egypt Public Health Assoc 2007 82 1–2 91 104 18217326

[B4] Afifi M Analysis of Saudi Medical Journal publications in PubMed, January 2001-November 2006 Saudi Med J 2007 28 7 1145 1147 17603734

[B5] Bakoush O Al-Tubuly A Ashammakhi N Elkhammas E PubMed Medical publications from Libya Libyan J Med 2007 2 3 125 128 10.4176/070625 21503210 PMC3078204

[B6] Bissar-Tadmouri N Tadmouri GO Bibliometric analyses of biomedical research outputs in Lebanon and the United Arab Emirates (1988–2007) Saudi Med J 2009 30 1 130 139 19139787

[B7] Deleu D Northway MG Hanssens Y Geographical distribution of biomedical publications from the Gulf Corporation Council countries Saudi Med J 2001 22 1 10 12 11255602

[B8] Page J Heller RF Kinlay S Lim LL Qian W Suping Z Kongpatanakul S Akhtar M Khedr S Macharia W Attitudes of developing world physicians to where medical research is performed and reported BMC Public Health 2003 3 6 10.1186/1471-2458-3-6 12529182 PMC149227

[B9] Rahman M Fukui T Biomedical publication–global profile and trend Public Health 2003 117 4 274 280 10.1016/S0033-3506(03)00068-4 12966750

[B10] Saravia NG Miranda JF Plumbing the brain drain Bull World Health Organ 2004 82 8 608 615 15375451 PMC2622935

[B11] Scopus SciVerse Scopus fact sheet 2012 SciVerse® Scopus, Elsevier B.V, Amsterdam, Netherlands http://www.info.sciverse.com/UserFiles/2508.SciVerse.Scopus_Facts_Figures%28LR%29.pdf. [Last accessed on 2012 Oct 19]

[B12] El Ansari W Afifi Soweid RA Jabbour S Geography of biomedical publications Lancet 2004 363 9407 489 490 14962532 10.1016/S0140-6736(04)15498-6

[B13] Hefler L Tempfer C Kainz C Geography of biomedical publications in the European Union, 1990–98 Lancet 1999 353 9167 1856 10.1016/S0140-6736(99)01278-7 10359422

[B14] Thompson DF Geography of U.S. biomedical publications, 1990 to 1997 N Engl J Med 1999 340 10 817 818 10075537 10.1056/NEJM199903113401020

[B15] Uthman OA Uthman MB Geography of Africa biomedical publications: an analysis of 1996–2005 PubMed papers Int J Health Geogr 2007 6 46 10.1186/1476-072X-6-46 17927837 PMC2098756

[B16] Tadmouri GO Bissar-Tadmouri N A major pitfall in the search strategy on PubMed Saudi Med J 2004 25 1 7 10 14758370

[B17] Falagas ME Pitsouni EI Malietzis GA Pappas G Comparison of PubMed, Scopus, Web of Science, and Google Scholar: strengths and weaknesses FASEB J 2008 22 2 338 342 17884971 10.1096/fj.07-9492LSF

[B18] de Granda-Orive JI Alonso-Arroyo A Roig-Vazquez F Which data base should we use for our literature analysis? Web of Science versus SCOPUS Arch Bronconeumol 2011 47 4 213 217 21281995 10.1016/j.arbres.2010.10.007

[B19] De Groote SL Raszewski R Coverage of Google Scholar, Scopus, and Web of Science: A case study of the h-index in nursing Nurs Outlook 2012 60 6 391 400 10.1016/j.outlook.2012.04.007 22748758

[B20] Kulkarni AV Aziz B Shams I Busse JW Comparisons of citations in Web of Science, Scopus, and Google Scholar for articles published in general medical journals JAMA 2009 302 10 1092 1096 10.1001/jama.2009.1307 19738094

[B21] Benamer H Bredan A Bakoush O The Libyan doctors’ brain drain: an exploratory study BMC Res Notes 2009 2 242 10.1186/1756-0500-2-242 19995446 PMC3225812

[B22] Benamer HT Bakoush O Medical education in Libya: the challenges Med Teach 2009 31 6 493 496 10.1080/01421590902832988 19811164

[B23] Benamer HT Bakoush O Arab nations lagging behind other Middle Eastern countries in biomedical research: a comparative study BMC Med Res Methodol 2009 9 26 10.1186/1471-2288-9-26 19374747 PMC2674457

[B24] Benamer HT Bredan A Bakoush O Scientific publication productivity of Libyan medical schools: a bibliometric study of papers listed in PubMed, 1988–2007 Educ Health (Abingdon) 2009 22 2 310 20029754

[B25] Benamer HT Bredan A Bakoush O A negative trend of biomedical research in Libya: a bibliometric study Health Info Libr J 2009 26 3 240 245 10.1111/j.1471-1842.2008.00792.x 19712216

[B26] Bredan A Benamer H Bakoush O Visibility of Arab countries in the world biomedical literature Libyan J Med 2011 6 6325 10.3402/ljm.v6i0.6325 PMC3081872 21526038

[B27] Tadmouri GO Biomedical science journals in the Arab world Saudi Med J 2004 25 10 1331 1336 15494797

[B28] Tadmouri GO Bissar-Tadmouri N Biomedical publications in an unstable region: the Arab world, 1988–2002 Lancet 2003 362 9397 1766 10.1016/S0140-6736(03)14868-4 14643139

[B29] Tadmouri GO Tadmouri NB Biomedical research in the Kingdom of Saudi Arabia (1982–2000) Saudi Med J 2002 23 1 20 24 11938358

[B30] Diab MM Taftaf RM Arabi M Research productivity in Syria: Quantitative and qualitative analysis of current status Avicenna J Med 2011 1 1 4 7 10.4103/2231-0770.83716 23210002 PMC3507054

